# Supplementary material for: Gut Bacterial Dysbiosis in Children with Intractable Epilepsy
Source: J Clin Med. 2020 Dec 22;10(1):5. doi: 10.3390/jcm10010005 (PMC7792797; doi:10.3390/jcm10010005)
Supplement: Supplementary file 1 [file jcm-10-00005-s001.pdf]

Supplementary Materials

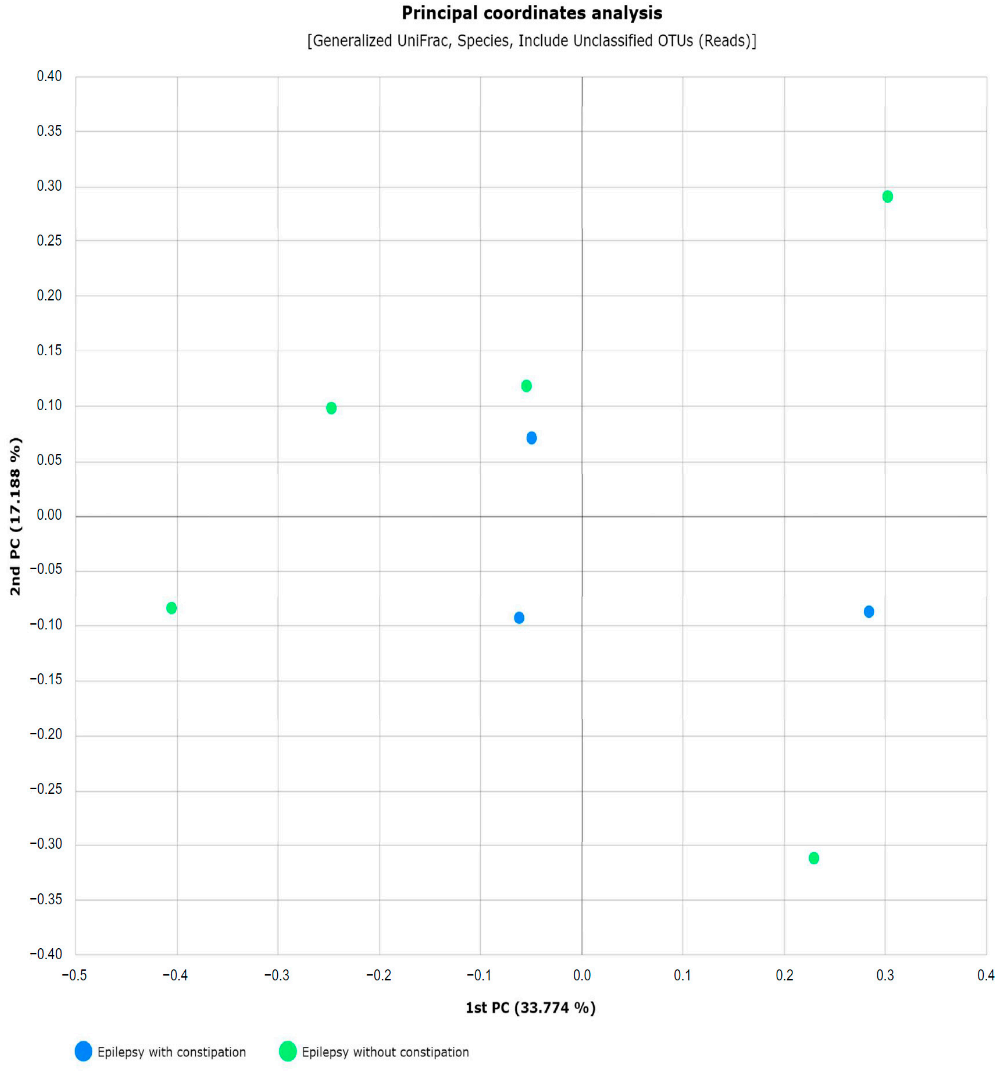

Figure S1. Comparison of gut bacterial taxonomic compositions of the patients with or without constipation. Principal coordinates were derived from the Unifrac distance among the subjects based on their species-level OTU compositions.

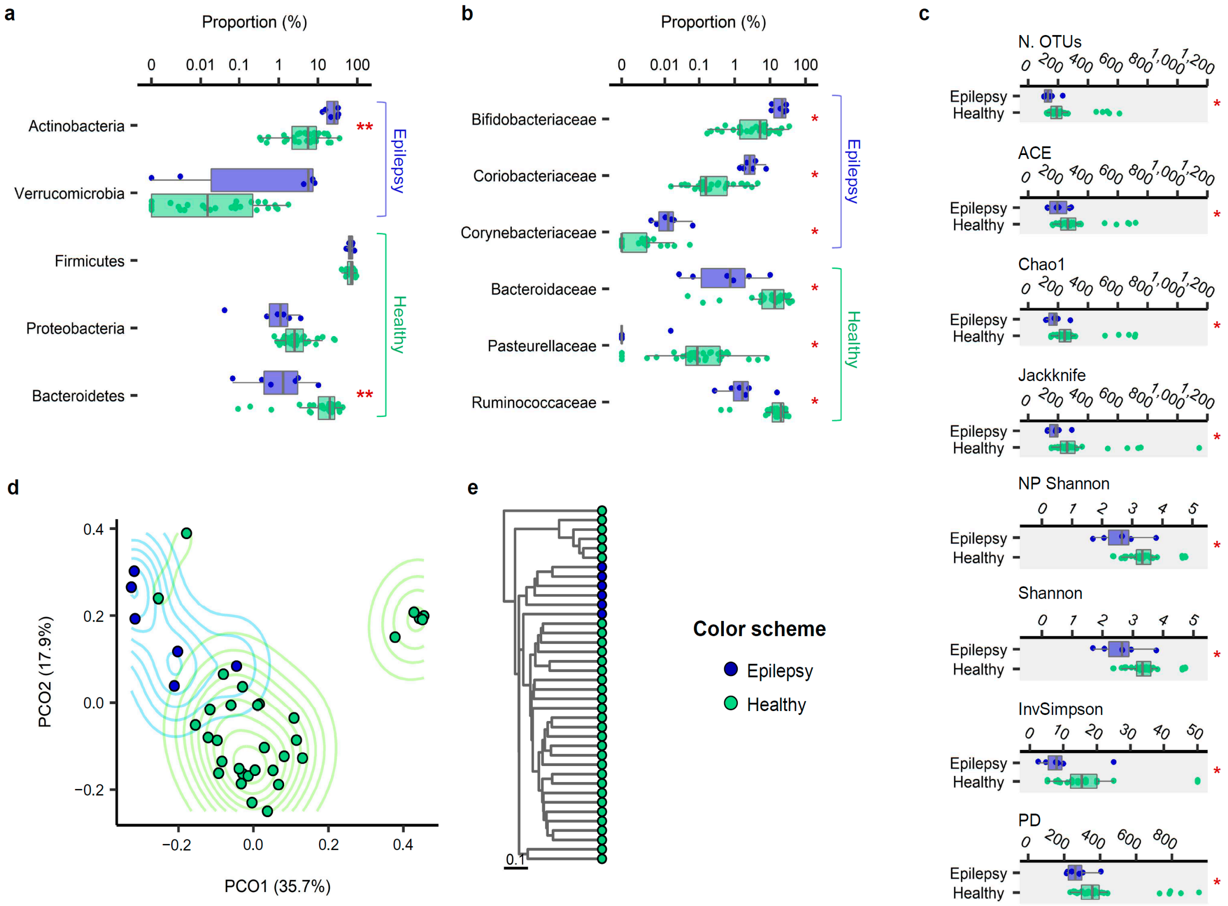

Figure S2. Community comparison repeated after excluding the subjects fed liquid diet. All the panels in the main Figure 1 were replicated here. (**a**) Phylum level compositions of gut microbiota compared between patients with intractable epilepsy and healthy controls, taxa displaying significant differences highlighted; (**b**) Comparison at family level; (**c**) Species richness and diversity index compared between patients with intractable epilepsy and healthy controls; (**d**) Principal coordinate analysis plot of the inter-subject variation of bacterial species-level OTU compositions based on UniFrac distance metric; (**e**) UPGMA dendrogram representing the species-level compositional distances among the subjects. * labeled to the comparisons with FDR < 0.05, ** labeled to p < 0.01, based on Mann-Whitney test and Benjamini-Hochberg adjustment for multiple comparisons.

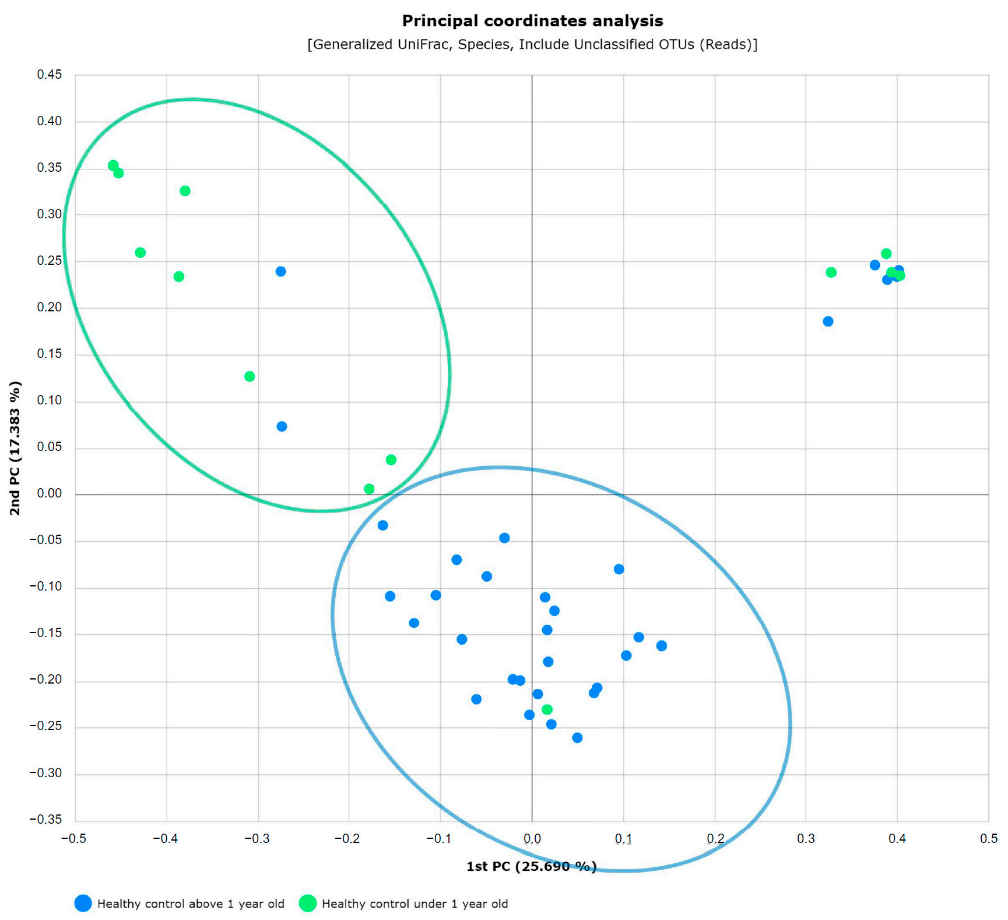

Figure S3. Gut bacterial community comparison between the subjects before and after 1 year of age. Principal coordinates were derived from the UniFrac distance among the species-level OTU composition of the subjects.

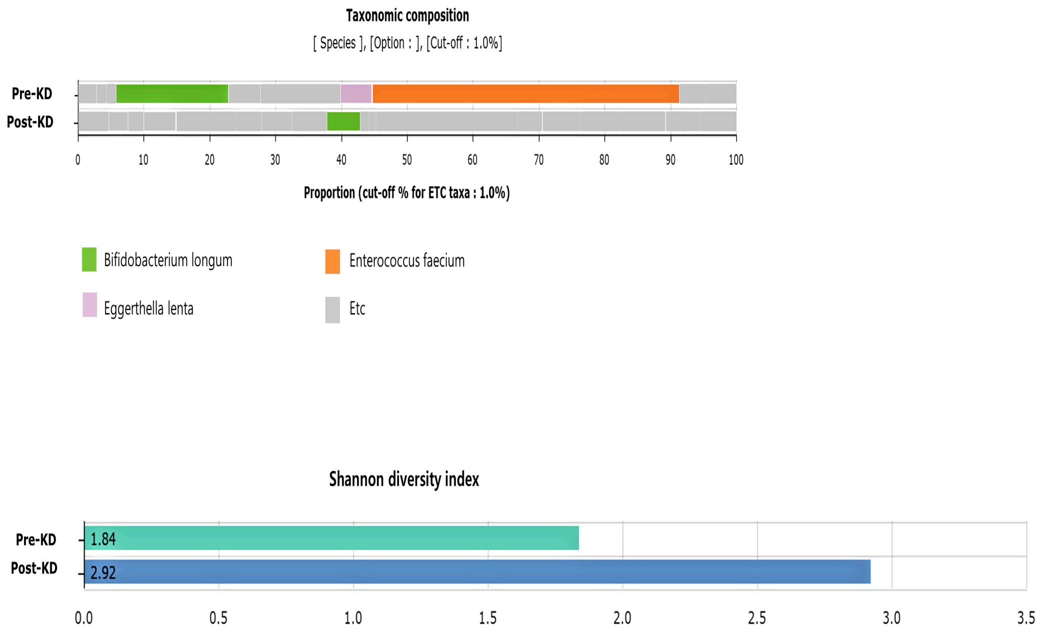

Figure S4. Changes in the gut microbiota after implementation of the ketogenic diet. A single patient was examined. In the taxonomic composition panel (upper) the epilepsy group-associated biomarkers discovered at species level were highlighted.

Table 1. Taxonomic differences depending on the presence of constipation in patients with epilepsy.

	Epilepsy with constipation (*n* = 3)	Epilepsy without constipation (*n* = 5)	Total (*n* = 8)	*p*
ACE	147.7 ± 36.9	184.9 ± 63.7	171.0 ± 55.5	0.400
Chao 1	147.3 ± 32.4	178.1 ± 64.1	166.5 ± 53.8	0.476
Jack knife	156.5 ± 34.2	192.1 ± 66.3	178.7 ± 56.5	0.430
NP Shannon	2.4 ± 0.5	2.3 ± 1.0	2.4 ± 0.8	0.944
Shannon	2.4 ± 0.5	2.3 ± 1.0	2.3 ± 0.8	0.942
Simpson	0.2 ± 0.1	0.2 ± 0.2	0.2 ± 0.2	0.554
Phylogenetic Diversity	252.7 ± 60.7	288.4 ± 99.2	275.0 ± 83.8	0.599

Abbreviations: ACE, abundance-based coverage estimator.
